# Critical Evaluation of Particle Size Distribution Models Using Soil Data Obtained with a Laser Diffraction Method

**DOI:** 10.1371/journal.pone.0125048

**Published:** 2015-04-30

**Authors:** Wang Weipeng, Liu Jianli, Zhao Bingzi, Zhang Jiabao, Li Xiaopeng, Yan Yifan

**Affiliations:** 1 State Key Laboratory of Soil and Sustainable Agriculture, Institute of Soil Science, Chinese Academy of Sciences, Nanjing, China; 2 Graduate University of Chinese Academy of Sciences, Beijing, China; Chinese Academy of Sciences, CHINA

## Abstract

Mathematical descriptions of classical particle size distribution (PSD) data are often used to estimate soil hydraulic properties. Laser diffraction methods (LDM) now provide more detailed PSD measurements, but deriving a function to characterize the entire range of particle sizes is a major challenge. The aim of this study was to compare the performance of eighteen PSD functions for fitting LDM data sets from a wide range of soil textures. These models include five lognormal models, five logistic models, four van Genuchten models, two Fredlund models, a logarithmic model, and an Andersson model. The fits were evaluated using Akaike’s information criterion (*AIC*), adjusted *R^2^*, and root-mean-square error (*RMSE*). The results indicated that the Fredlund models (FRED3 and FRED4) had the best performance for most of the soils studied, followed by one logistic growth function extension model (MLOG3) and three lognormal models (ONLG3, ORLG3, and SHCA3). The performance of most PSD models was better for soils with higher silt content and poorer for soils with higher clay and sand content. The FRED4 model best described the PSD of clay, silty clay, clay loam, silty clay loam, silty loam, loam, and sandy loam, whereas FRED3, MLOG3, ONLG3, ORLG3, and SHCA3 showed better performance for most soils studied.

## Introduction

Modeling the size distribution of soil particles to obtain a continuous particle size distribution (PSD) curves is useful for understanding essential soil properties such as pore distribution, water retention, hydraulic conductivity, and thermal and adsorption properties [1–4]. However, conventional PSD analysis captures only a limited number of particle mass fractions. Therefore, to be able to use these discrete experimental PSD data to estimate other soil properties, many researchers have used parametric functions to extend the limited scope of PSD data.

Soil PSD is frequently assumed to follow a lognormal distribution [5, 6], although some soils do have a bimodal PSD [7]. Buchan and Hwang compared five lognormal models and certain other types of models in terms of their fit to experimental PSD data [8, 9]. Their results showed that the bimodal model gave a marginally better fit, but incorporates a sub-clay mode, and the Jaky model gave a good fit to data for many soils, better than the standard lognormal model for 23 soils. The latter included models based on water-retention curves [10–12], the fragmentation model [13], power-law functions based on fractal geometry [13, 14], exponential functions [15], Gompertz equations [16], and a model estimating PSD from limited soil texture data [17].

Many researchers have relied upon a single PSD model at one time to represent a wide range of soil texture classes, although several studies have suggested that the performance of PSD models can be affected by soil texture [18, 9]. Buchan [5] proposed that lognormal models are adequate to describe only half the United States Department of Agriculture (USDA)’s soil texture triangle, and more complex models are needed to depict other soils such as sandy clay loam, sandy clay, and most clays. Rousseva [15] proposed and investigated the suitability of exponential and power-law distribution models to fit PSDs with diverse shapes and varying numbers of measured points. The results showed that model performance was affected by soil texture, but that these functions were a good fit for the PSD of both fine-and coarse-textured soils. Fredlund [10] pointed out that the suitability of their own model improved as the proportion of clay in soils increased. Contrasting results have been obtained for the fitting of some models. For instance, in some cases, the Skaggs model performed poorly for silty soils [17], whereas in other cases, the same model performed better when silt content was higher [18].

Soil PSD data in most databases are obtained via the sieve-pipette method (SPM) based on Stokes’s law [19]. This traditional approach is a tedious and time-consuming procedure and does not adequately describe soil PSD, especially for the clay fraction (<2 μm) [20]. As a faster alternative, laser diffraction method (LDM) techniques have been increasingly used recently because they require a much smaller sample and provide highly reproducible PSD results, encompassing a broader range of size classes than conventional methods [21–24]. The LDM was reported to underestimate the clay fraction and overestimate the silt fraction of soils compared to the SPM [25]. Some comparative studies of PSD functions with various underlying assumptions have been performed, mostly with sedimentation data sets [26, 8, 18, 9]. In a comparison of several PSD models with PSD data obtained using both LDM and SPM approaches, fractal and exponential functions fitted poorly, whereas the suitability of the Gompertz model increased with clay content for the LDM data sets [26]. In the same analysis, the Fredlund function provided very good fits with SPM-derived PSD, but not with the corresponding LDM data sets. The lognormal function showed better overall performance and provided very good fits with both SPM and LDM data sets. However, Buchan [5] found that a lognormal model could adequately describe only about half the United States Department of Agriculture (USDA)’s texture triangle

Few comparisons have been performed of the fitting performance of PSD functions based on LDM data sets. Furthermore, the effect of texture on PSD model performance has not been fully investigated. Therefore, the objectives of this study were to (1) use a variety of models with different underlying assumptions to test the fit of varying PSD functions to laser-derived PSD of soils from across China, and (2) to determine whether soil texture significantly affects model performance. Three statistical indices were used to define the best models: the adjusted root-mean-square error (*RMSE*), the adjusted *R* ^*2*^, and Akaike’s information criterion (*AIC*).

## Materials and Methods

### Soil samples

i)No specific permissions were required for these sampling points; ii) these field studies did not involve endangered or protected species.

**Fig 1:**
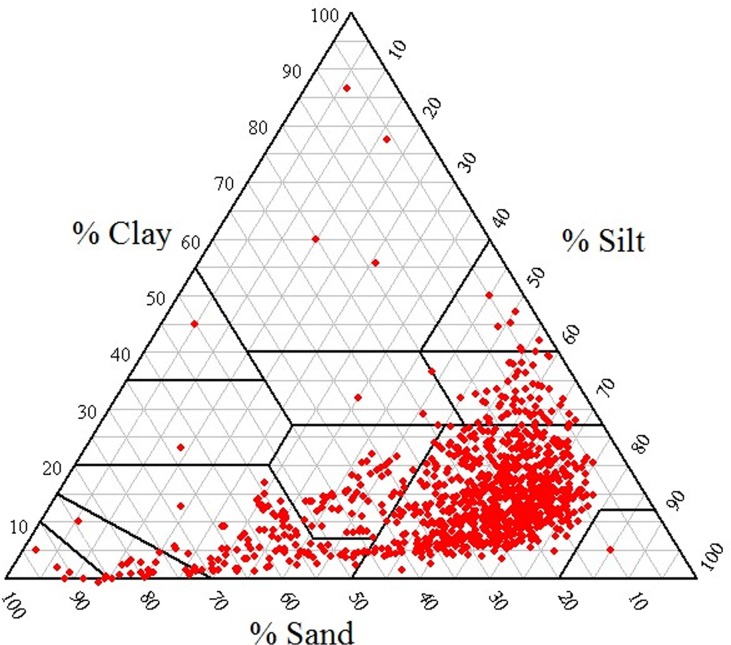
Textural composition of soil samples.

Samples of 1013 soils were collected from 13 provinces of China, representing a diversity of parent materials, climates, and times of soil formation and considerable variance in soil PSD, texture (Fig 1), and mineralogy. The sampling locations were as follows: 96 soil profiles were taken from Yellow River flood areas (Fengqiu, Henan), with samples collected every 10 cm in each profile from depths of 0–200 cm, for a sub-total of 960 samples; 6 samples were obtained from paddy soils in the Taihu Lake region (Changshu, Jiangsu); and 47 soil samples were obtained from 10 different provinces in China (Table 1). Samples were collected, air-dried, gently crushed, and dry-sieved through a 2-mm mesh to remove coarse fragments. Hydrogen peroxide at 30% concentration was used to remove the organic materials.

**Table 1:** General characteristics of the different types of soils studied.

Location	Classification ^1^	Parent Material	SPM (%)			Clay mineral composition^2^
			Clay	Silt	Sand	
Fengqiu, Henan	Durudepts	Yellow River alluvial	9.68–18.56	12.57–40.32	28.62–79.58	Quartz, Hydromica, FeldsparChlorite
Fengqiu, Henan	Haplustepts	Yellow River alluvial	4.26–25.36	9.28–53.35	20.82–86.48	Quartz, Hydromica, FeldsparChlorite
Fengqiu, Henan	Fragiudepts	Yellow River alluvial	5.86–29.66	6.27–55.15	25.82–89.48	Quartz, Hydromica, FeldsparChlorite
Changshu, Jiangsu	Aquanthrepts	Lacustrine deposits	27.16	47.58	25.26	Quartz, Feldspar Hydromica
Changshu, Jiangsu	Aquanthrepts	Lacustrine deposits	9.60	55.45	34.95	Quartz, Feldspar Hydromica
Changshu, Jiangsu	Aquanthrepts	Lacustrine deposits	26.00	45.70	28.30	Quartz, Feldspar Hydromica
Changshu, Jiangsu	Aquanthrepts	Lacustrine deposits	21.10	51.60	27.30	Quartz, Feldspar Hydromica
Changshu, Jiangsu	Aquanthrepts	Lacustrine deposits	20.59	47.59	31.81	Quartz, Feldspar Hydromica
Changshu, Jiangsu	Aquanthrepts	Lacustrine deposits	25.10	48.60	26.30	Quartz, Feldspar Hydromica
Nanjing, Jiangsu	Aquanthrepts	Xia—Shu loess	27.46	46.94	25.60	illite, Kaolinite, Vermiculite
Nanjing, Jiangsu	Aquanthrepts	Xia—Shu loess	18.27	46.91	34.82	illite, Kaolinite, Vermiculite
Yingtan, Jiangxi	Rhodudults	Red sandstone	39.97	27.17	32.86	Quartz, Montmorillonite, Vermiculite,
Yingtan, Jiangxi	Sulfudepts	Red sandstone	24.30	38.80	36.90	Quartz, Montmorillonite,Kaolinite
Yingtan, Jiangxi	Sulfudepts	Red sandstone	21.10	43.80	35.10	Quartz, Montmorillonite,Kaolinite
Yingtan, Jiangxi	Hapludults	Red sandstone	10.81	11.35	77.84	Quartz, Montmorillonite,Kaolinite
Shantou, Guangdong	Rhodudults	Granite	33.30	31.26	35.44	Quartz, Hydromica, Chlorite
Shenzhen, Guangdong	Hapludox	Granite	15.72	19.43	64.85	Quartz, Hydromica, Kaolinite
Binhai, Tianjin	Haplaquepts	Marine sediments	39.18	35.38	25.45	illite,Montmor-illonite, Chlorite
Urumuqi, Xinjiang	Calciargids	Proluvium	18.72	31.83	49.45	illite, Chlorite, Kaolinite
Yanglin, Shanxi	Plagganthrepts	Allochthonous soil	16.26	49.46	34.28	illite, Montmorillo-nite, Vermiculite
Beibei, Chongqing	Pupliudepts	Jurassic Period Zisha Rock	29.97	34.95	35.08	Vermiculite, Kaolinite
Haikou, Hainan	Acraquox	Basalt	59.73	18.58	21.69	Kaolinite, Gibbsite, Quartz
Wencang, Hainan	Acrudox	Basalt	64.14	14.61	21.25	Kaolinite, Gibbsite, Quartz
Nehe, Heilongjiang	Argiaquolls	Proluvium	39.07	38.27	22.66	Montmorillonite, Hydromica, Feldspar
Binxiang, Heilongjiang	Frigudepts	Granitic gneiss	33.85	44.18	21.97	Montmorillonite, Hydromica, Feldspar
Shenyang, Liaoning	Natrudalfs	Quaternary Malan loess	20.05	32.12	47.82	Montmorillonite, Hydromica, Kaolinite
Kunming, Yunnan	Fragiudults	Argillaceous rock	52.89	19.04	28.08	Kaolinite, Gibbsite
Ningbo, Zhejiang	Halaquepts	Marine sediments	11.40	54.30	34.30	illite, Kaolinite, Vermiculite

SPM = sieve-pipette method;

^1^USDA = United States Department of Agriculture; NRCS = National Research Council Service;

Classification: the soils were classified according to the USDA, NRCS: Keys to Soil Taxonomy, Eleventh Edition.

^2^A semi-quantitative result of soil mineral composition by X-ray diffraction patterns.

### Apparatus and parameter settings for the LDM

The main illumination source for PSD analysis in a Beckman-Coulter LS-13 320 was a 5 mW laser diode (wavelength: 750 nm). The analyzer provided PSD in the form of 93 bins within the diameter range of 0.38–2000 μm (19 bins in the range of 0.38–2 μm and 74 bins in the range of 2–2000 μm). Because the LD analyzer required a very small subsample (<5 g) for the PSD determination by LDM, each sample was blended several times before testing to minimize the potential variability of the results caused by differences between subsamples. Soil samples (5 g, <2 mm) received the same pretreatment as for the SPM [19]; particles were then transferred to 100-mL cylinders containing 10 mL of a 0.5 mol L^-1^ sodium hexametaphosphate (for soils with pH >7.5), sodium oxalate (soil pH = 6.5–7.5), or sodium hydroxide (soil pH <5.5) solution. The samples were left overnight to disperse, then transferred to fluid modules and diluted to 10% obscuration [27]. Each sample was subjected to a 60-sec optical measurement [2]. A soil particle refractive index of 1.50 and a soil particle absorption index of 0.01 were regulated for the Mie theory model [24].

### Particle Size Distribution Functions

Eighteen PSD functions with different underlying assumptions were evaluated using PSD data derived from the procedures described above (Table 2). The five models were a Jaky function (JAKY1, with the number representing the quantity of model parameters) [28]; a simple lognormal (LOGN2) [5]; an offset non-renormalized lognormal model (ONLG3) [8]; an offset renormalized lognormal model (ORLG3) [8]; and a Shiozawa-Campbell model (SHCA3) all of which assume that the soil PSD follows a lognormal pattern [29]. In the JAKY1 model, *p* represented the PSD index that describes the stretching of the curve, and *d*_*0*_ was the maximum diameter (see equations in Table 2). In LOGN2, ONLG3, ORLG3, and SHCA3, *F*_*n*_ was the cumulative normal distribution function, *μ* was the mean, and *σ* was the standard deviation [8]. For the logarithmic function (LOGL2) as used by Zhuang to generate detailed PSD for fine-textured soils, *a* and *b* were fitting parameters [30]. The five logistic models a logistic growth model (LOGI2) [17], and three logistic growth model extension models, LOGI3, MLOGI3 and LOGI4, along with the Gompertz function (GOMP4) [16] were based on the assumption that the soil PSD followed a logarithmic growth function. Based on the LOGI2 model, the LOGI3, MLOGI3, and LOGI4 models were generated by respectively altering, adding, or reducing the parameters of the original LOGI2. The main procedure used for this extension was the inclusion of one or more adaptation coefficients to replace the constants in the original formula (see Eqs 1 through 4 below). GOMP4 was a logistic function represented by a closed-form equation, with *α*, *β*, *γ*, and *μ* being the shape parameters of the curve. The four van Genuchten type models [31] the Havrkamp-Parlang model (VANH2) [11], and the VANH2 extension models (VANZ2, VANZ3, and VANG4; see Eqs 5–8 below) were derived from the van Genuchten function that represents the water-retention characteristics of soils. This approach was based mainly on the similarity between PSD shapes and water-retention curves. The Fredlund functions (FRED3, FRED4) and the Andersson model were also based on the similarity between soil water-retention and PSD curves [10]. In the FRED3 and FRED4 models, *α* was the point of inflection of the curve, *n* was related to its steepest slope, *m* to its shape near the fine region, *d*_*f*_ was the number of fine particles, and *d*_*m*_ was the minimum particle size. Notably, FRED3 was a special case of FRED4 (*d*_*f*_ = 0.01). In the Andersson model, *α*, *β*, were shape parameters, and *d*_*0*_ denoted the most frequent particle diameter corresponding to the cumulative percent *d*_*f1*_ [32, 33]. Because of the limited number of classes in the clay range, even with the LDM (0.5 and 1 μm), the bimodal model was not evaluated in this study. All models were fitted to experimental PSD data for the 1013 sampled soils using an iterative nonlinear regression procedure which found the values of the fitting parameters that gave the best fit between the model and the data. This procedure was performed using the modified nonlinear least-squares method (MNLS) [34].

**Table 2:** Particle size distribution (PSD) functions used in the study.

Model N°.	Abbreviation^1^	Model type and Author	Equation^2^	Parameters^3^
1	*JAKY1*	*Jacky* [28]	*F*(*d*) = *exp* {−1/*p*^2 [*ln*(*d*/*d*_0)]^2}	*p*
2	*LOGL2*	*Logarithmic* [30]	*F*(*d*) = *a* ln *d* + *b*	a, b
3	*LOGI2*	*Logistic growth model* [17]	*F*(*d*) = 1/(1 + *α* exp(− *β d*))	α, β
4	LOGN2	Simple lognormal [5]	$\begin{array}{l} F_{1}\left(x\right)=\left(1+\;erf\;\left[\left(x-\mu_{1}\right)/\sigma_{1}\;\sqrt{2}\right]\right)/2\;\left(x\;\geq \;\mu_{1}\right)\\ F_{2}\left(x\right)=\left(1-erf\;\left[\left(x-\mu_{1}\right)/\sigma_{1}\;\sqrt{2}\right]\right)/2\;(x<\;\mu_{1})\\ x\;=ln\left(d\right) \end{array}$	μ, σ
5	VANH2	Haverkamp—Parlange [11]	$F\left(d\right)=1/{\left[\left(1+\frac{\alpha^{\beta }}{d}\right)\right]}^{1-1/\beta }$	α, β
6	VANZ2	Modified van Genuchten [30–31]	$\text{F}\left(d\right)\;=\;d_{min}\;+\;\left(1-\;d_{min}\right)\;{\left[1+{\left(\alpha d\right)}^{\beta }\right]}^{-1+\frac{1}{\beta }}$	α,β, d_min_
7	FRED3	Fredlund [10]	$F\left(d\right)=\frac{1}{{\left\{ln\left[exp\left(1\right)+{\left(\frac{\alpha }{d}\right)}^{n}\right]\right\}}^{m}}\;\left\{1-\;{\left[\frac{ln\left(\;1+\frac{1}{d}\right)}{ln\left(1+\frac{0.01}{d_{min}}\right)}\right]}^{7}\right\}$	α, m n
8	LOGI3	Logistic growth function extension model 1	*F*(*d*) = *ε*/(1 + *α exp*(− *β d*))	α, β, ε
9	MLOGI3	Modified logistic growth model	*F*(*d*) = 1/[1 + *α exp*(− *βd* ^*ϵ*^)]	α, β, ε
10	ONLG3	Offset Non-renormalized Lognormal [8]	*G*(*x*) = *F*(*x*) + *c*_1_ *x* = *ln d F(X)* defined by *LOGN2* model.	μ, σ, c_1_
11	ORLG3	Offset Renormalized Lognormal [8]	*G*(*x*) = (1 – *c*_2_)*F*(*x*) + *c*_2_ *x* = *ln d F(X)* defined by *LOGN2* model.	μ, σ, c_2_
12	SHCA3	Shiozawa-Campbell [3]	$G\left(x\right)=\varepsilon F_{1}\left(x\right)+\left(1-\varepsilon \right)F_{2}\left(x\right)\;F_{1}\left(x\right)=\left(1+erf\left[\left(x-\mu_{1}\right)/\sigma_{1}\;\sqrt{2}\right]/2\right)\;\left(x\geq \mu_{1}\right)\;F_{2}\left(x\right)=\left(1-erf\left[\left(x-\mu_{1}\right)/\sigma_{1}\;\sqrt{2}\right]/2\right)\;(x<\mu_{1})\;x=ln\;\left(d\right)$	σ_1_, μ_1_
13	VANZ3	van Genuchten, function extension model 1	$F\left(d\right)\;=\;\varepsilon +\;\left(1-\varepsilon \right)\;{\left[1+{\left(\alpha d\right)}^{\beta }\right]}^{-1+\frac{1}{\beta }}$	α, β, ε
14	ANDE4	Andersson [32]	$F\left(d\right)\;=\;d_{f1}+\;\alpha \;{tan}^{-1}(\beta \;log\frac{d}{d_{0}})$	α, β, d_f1_,d_0_
15	FRED4	Fredlund [10]	$F\left(d\right)=\frac{1}{{\left\{ln\;\left[exp\left(1\right)+{\left(\frac{\alpha }{\text{d}}\right)}^{n}\right]\right\}}^{m}}\;\left\{1-\;{\left[\frac{\text{ln}\left(\;1+\frac{d_{f}}{d}\right)}{\text{ln}\left(\;1+\frac{d_{f}}{d_{min}}\right)}\right]}^{7}\right\}$	α, n, m, d_f,_ d_min_
16	GOMP4	Gompterz [16]	*F*(*d*) = *α* + *γ exp*{−*exp*[−*β*(*d* – *μ*)]}	α, β, γ, μ
17	LOGI4	Logistic growth function extension model 2	*F*(*d*) = *β* + (*α* − *β*)/(1 + (*d*/*ε*)^*γ*)	α, β, ε, γ
18	VANG4	van Genuchten, function extension model 2	$F\left(d\right)\;=\;\varepsilon +\;\left(\gamma -\;\varepsilon \right){\left[\gamma +{\left(\alpha d\right)}^{\beta }\right]}^{-1+\frac{1}{\beta }}$	α, β, ε, γ

^1^The number in the model abbreviation represents the number of the function parameters.

^2^ *d*, particle diameter in mm. Erf, error function

^*3*^ *p*: characterizes the stretching of the PSD curve; *d*_*0*_ = 2 mm; *d*_*f*_, number of fine particles; *d*_*f1*_, number of *d*_*0*_; *d*_*min*_ = 0.001 mm. *d*_*0*_, diameter of the most frequent particle. *a* and *b*, fitting parameters; *c*_*1*,_ *c*_*2*_: offset parameters.*α*, *β*, *m* and *n*: shape parameters; *μ*, mean; *σ*, standard deviation; *d*_*min*_: minimum diameter; *ε* and γ fitting parameters. *σ*_*1*_ = 1.00; *μ*_*1*_ = -1.96.

### The logistic growth model and its extensions

The logistic growth function [35] is widely used in studies involving forecasting of biotic populations and system reliability analysis. When used to depict soil PSD, the model is modified as follows:

$$F\left(d\right)=\frac{1}{1+\alpha \;exp\left(-\;\beta \;d\right)}\;,$$ (1)

*α*, *β*: shape parameters (α > 0, β > 0).

To improve the fitting ability of LOGI2, a fitting coefficient *ε* was used as a substitute for the LOGI2 numerator constant “1”; in this way, the logistic growth function extension model LOGI3 was obtained. This model can be expressed as follows:

$$F\left(d\right)=\frac{\varepsilon }{1+\;\alpha \;exp\left(-\;\beta \;d\right)}\;,$$ (2)

*α*, *β*: shape parameters (α >0, β > 0); *ε*: fitting parameter.

Based on the LOGI2 model, the MLOG3 model was generated by adding an index parameter *c* to Equation [1] as follows:

$$F\left(d\right)=\frac{1}{1+\;\alpha \;\text{exp}\left(-\beta d^{c}\right)}\;,$$ (3)

*α*, *β*: shape parameters (*α* >0, *β* > 0); *c*: fitting parameter.

Similarly, the LOGI4 model was obtained by adjusting the parameters of the MLOG3 model and rewriting Equation [3] as:

$$F\;\left(d\right)=\;\beta \;+\frac{\alpha -\;\beta }{1+\;{\left(\frac{d}{\varepsilon }\right)}^{\gamma }}\;,$$ (4)

*α*, *β*: shape parameters (*α* >0, *β* > 0); *ε*, *γ*: fitting parameters.

### The van Genuchten model and its extensions

The van Genuchten model (VANH2) was developed initially to depict soil moisture characteristic curves. To use it to depict soil PSD, the original function was modified as follows:

$$F\;\left(d\right)=\frac{1}{{\left[1+{\frac{\alpha }{d}}^{\beta }\right]}^{1-\frac{1}{\beta }}}\;,$$ (5)

*α*, *β*: shape parameters.

Based on the VANH2 model, Zhuang modified the van Genuchten model and used the extension model (VANZ2) for soil PSD as follows [30]:

$$F\;\left(d\right)\;=\;d_{min}\;+\;\left(1-\;d_{min}\right){\left[1+{\left(\alpha d\right)}^{\beta }\right]}^{-1+\frac{1}{\beta }}\;,$$ (6)

*α*, *β*: shape parameters; *d*_*min*_: minimum diameter.

In addition, by substituting the fitting parameter ‘*ε*’ for the *d*_*min*_ of Equation [6], the generic form of the VANH2 model can be obtained. This van Genuchten extension model was called VANZ3 and defined as follows:

$$F\;\left(d\right)\;=\;\varepsilon +\;\left(1-\;\varepsilon \right)\;{\left[1+{\left(\alpha d\right)}^{\beta }\right]}^{-1+\frac{1}{\beta }}\;,$$ (7)

*α*, *β*: shape parameters; *ε*: fitting parameter.

Again, using a fitting parameter *γ* to replace the constant *1* of Equation [7], four-parameter van Genuchten model (VANG4) was obtained as follows:

$$F\;\left(d\right)\;=\;\varepsilon +\;\left(\gamma -\;\varepsilon \right)\;{\left[\gamma +{\left(\alpha d\right)}^{\beta }\right]}^{-1+\frac{1}{\beta }}\;,$$ (8)

*α*, *β*: shape parameters; *ε*, *γ*: fitting parameters.

### Model Comparison and Fitting Techniques

Several approaches have been reported for choosing the most suitable model. The simplest approach is to find the model that minimizes the discrepancy between observed and predicted data. However, it must be recognized that as the number of fitted parameters increases, the fitting performance generally improves. This occurs at the cost of a corresponding increase in the probability of over parameterization. The PSD models considered here required between one and four fitting parameters. To minimize the risk of “over fitting” models with too many parameters, a better approach was to define the optimum model as the one that fitted the data well with the least number of fitting parameters, all other conditions being equal. Several researchers have used this kind of criterion to select the best model. *AIC* [36] has been frequently used to identify the best predictive function for soil moisture characteristics because it can be determined indirectly from other easily measured soil properties [37], and to select the best soil hydraulic function [9, 18, 26].

The correspondence of model predictions with experimental data was ascertained using the adjusted root mean square error (*RMSE*) and *R* ^*2*^ because of the unequal number of parameters in the PSD functions:

$$RMSE\;\left(adjusted\right)=\sqrt{\frac{SSE}{n-p}}\;,$$ (9)

$$R^{2}\;\left(adjusted\right)\;=\;1-\left[\frac{SSE/\left(n-p\right)}{SST/\left(n-1\right)}\right]\;,$$ (10)

where *SSE* is the sum of squared errors, *SST* the total sum of squares, *n* the total number of PSD data points, *P* the number of model parameters, and

$$SSE\;=\sum_{i=1}^{n}{\left(Y_{m}-Y_{c}\right)}^{2},$$ (11)

*Y*_*m*:_ measured; Yc: estimated.

*AIC* values were used to compare the various models. According to this criterion, the model with the smallest *AIC* value was selected as the best soil description. *AIC* imposes a penalty for additional fitting parameters, which was calculated as:

AIC=Nln(SSE)+2P. (12)

### Theory for verifying the precision of the LDM PSD

As pointed out by Miller and Schaetzl [38], the cumulative bin difference (*CBD*) ^(Eq.13)^ plus one standard deviation (*SD*) can be used as a precision threshold (PH) ^(Eq.14)^ to verify the measurement variability among the subsamples. In this way, the data from the first two subsamples were used as a starting point to ascertain the normal amount of intra-sample variation. The *CBD* was larger when many bins had small differences, as in the LDM analysis which provided a PSD with 117 bins. The *CBD* was calculated by adding the absolute differences between the two subsamples for all bins. The definition of the *CBD* was as follows:

$$CBD=\sum_{k=1}^{n}\left|a_{k}-b_{k}\right|,$$ (13)

where *a* is the first subsample value, *b* is the second subsample value, and *n* is the number of bins, and that of *PH* was:

$$PH=\frac{\sum_{1}^{m}CBD}{m}\;+\;SD,$$ (14)

where *PH* is the precision threshold, *m* is the number of samples, and *SD* is the standard deviation for *CBD*s of all samples.

In the present study, *PH* was used to identify outliers. When a set of subsample pairs had a *CBD* exceeding 1 *SD* plus the mean *CBD* of all samples, the precision or repeatability of the PSD results were considered insufficient for this type of analysis. In these cases, additional subsamples were measured using the same procedure as for the previous subsamples until every sample had a pair of subsamples with a *CBD* less than the *PH*, which meant that some samples had to undergo more than two analyses to obtain an eligible *CBD*. The samples chosen for additional measurements were only those that continued to have a *CBD* greater than *PH*. For those samples which had been measured several times, the mean of the two subsample values with the least *CBD* was used to represent the PSD for each sample.

## Results and Discussion

LDM provides a rapid solution for obtaining soil PSD, especially when analyzing a large number of soil samples. However, there are disadvantages associated with the use of LDM for PSD measurements over the traditional SPM. In this study, the LDM underestimated the clay fraction by as much as 35% and overestimated the silt fraction by about 27% compared with SPM (data not shown). This is consistent with results obtained elsewhere [22, 23, 25]. These differences are attributable to the heterogeneity of soil particle density and the deviation of particle shapes from sphericity. Sedimentation methods assume a single particle density, which is a major source of error, whereas LDM is independent of particle density. Deviation from sphericity, however, is the major factor that has disparate effects on both methods. In the case of LDM, an irregularly shaped soil particle reflects a cross-sectional area greater than that of a sphere of equivalent volume [25]. Therefore, particles tend to be assigned to larger size fractions of the PSD, thereby resulting in a smaller clay fraction. On the other hand, nonspherical particles in the pipette method have longer settling times than their equivalent spheres, resulting in overestimation of the clay fraction.

### Definition of the optimum model

**Fig 2:**
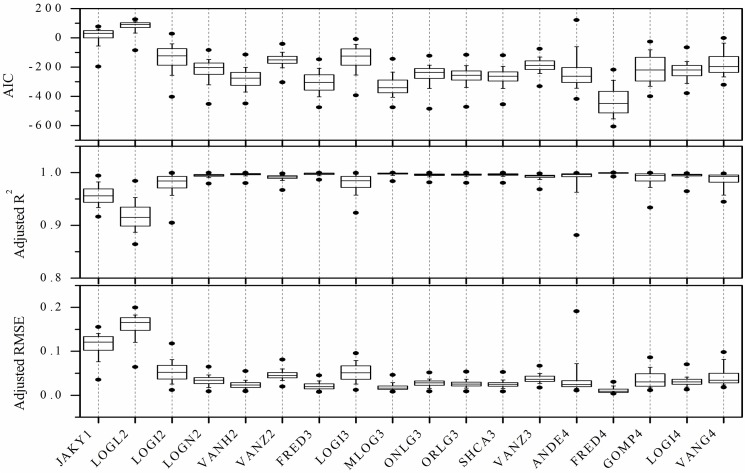
Performance of eighteen mathematical functions fitted to ninety-three class particle size distributions (PSD) based on soil data obtained with laser diffraction methods (LDM), in terms of Akaike’s information criterion (*AIC*), adjusted R^2^, and adjusted root mean square error (*RMSE*).

**Fig 3:**
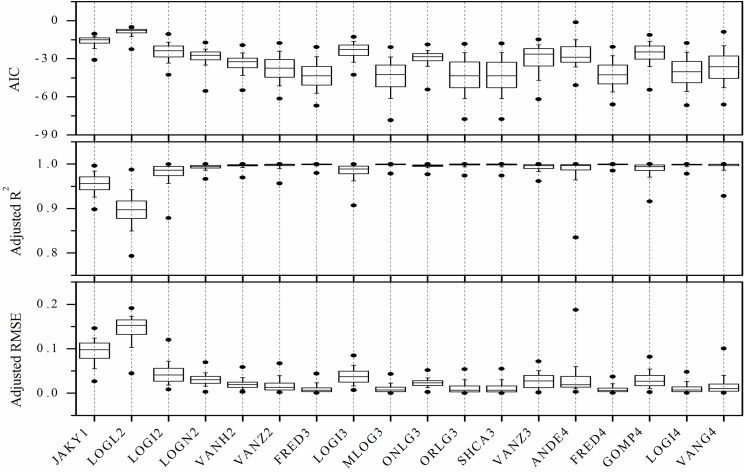
Performance of eighteen mathematical functions fitted to six-class (2, 20, 50, 125, 250 and 1000 μm) particle size distributions (PSD) based on soil data obtained with the laser diffraction method (LDM), in terms of Akaike’s information criterion (*AIC*), adjusted R^2^, and adjusted root mean square error (*RMSE*).

Given the inherent limitation of PSD based on SPM—that the soil PSD becomes characterized by limited particle size data—this study verified the performance of the mathematical models using SPM PSD results with a different data set. Each soil sample’s six classes (2, 20, 50, 125, 250, and 1000 μm) of the LDM PSD data were extracted to construct the comparison LDM PSD set. Results for model fitting of the PSD functions to the data sets are shown in Figs 2 and 3. Overall, *AIC* values ranged from -642.432 to 154.635 (in a 93-class PSD) and from -0.382 to -87.286(in a six-class PSD data). The adjusted *RMSE* obtained for regression of estimated on measured cumulative volume fractions ranged from 0 to 0.233 (for 93-class PSD data) and from 0 to 0.205 (for six-class PSD data). With adjustments to *R* ^*2*^ values among models and between groups, the differences were somewhat less than what had been noted for *AIC* values and adjusted *RMSE* values (S1 and S2 Datasets).

Low *AIC*, low adjusted *RMSE*, and high adjusted *R* ^*2*^ indicate better correspondence of a model with experimental data, and vice versa. Based on the performance of eighteen models with the 93-class PSD data (Fig 2), the models were ranked in order from best to worst fit, according to the comparison criteria: FRED4 > MLOG3 > FRED3 > VANH2 > SHCA3 > ORLG3 > ONLG3 > ANDE4 > LOGI4 > LOGN2 > VANZ3 > VANG4 > VANZ2 > GOMP4 > LOGI2 > LOGI3 > JAKY1> LogL2. Correspondingly, for the comparison data set, the sequence of model suitability was: FRED4 > FRED3 > MLOG3 > ORLG3> SHCA3 > LOGI4 >VANZ2 > VANG4 > VANH2 > ONLG3 > LOGN2 > VANZ3 > ANDE4 > GOMP4 > LOGI2 > LOGI3 > JAKY1 > LogL2.

The lowest adjusted *RMSE* values for both the 93-class and the 6-class PSD data, indicating best fit, were obtained with the FRED4 function, followed by the MLOGI3 or FRED3 functions. Comparatively, the worst fits were obtained with the LOGL2 function, followed by the JAKY1, LOGI3, LOGI2, and GOMP4 functions. ONLG3 sometimes showed similar fitting performance to the ORLG3 and SHCA3 functions. As expected, the PSD functions other than JAKY1 and LOGL2 gave relatively better fits with the 93-bin PSD data compared to those with the comparison set of 6-bin PSD data. These differences in fitting ability may originate from the difference of data density between the two groups’ PSD models; in other words for the 6-bin PSD data, the comparison set’s PSD curve had longer between-point distances than the curve for soils with the ninety-three bin PSD data. This difference may render it impractical to construct a soil PSD accumulation distribution curve with an “actual shape”. Fortunately, the relative performance among the different models in the two groups was basically consistent, which means that the experimental results for the two data groups can be used to analyze the actual effect of using these PSD models to derive continuous soil PSD curves.

**Fig 4:**
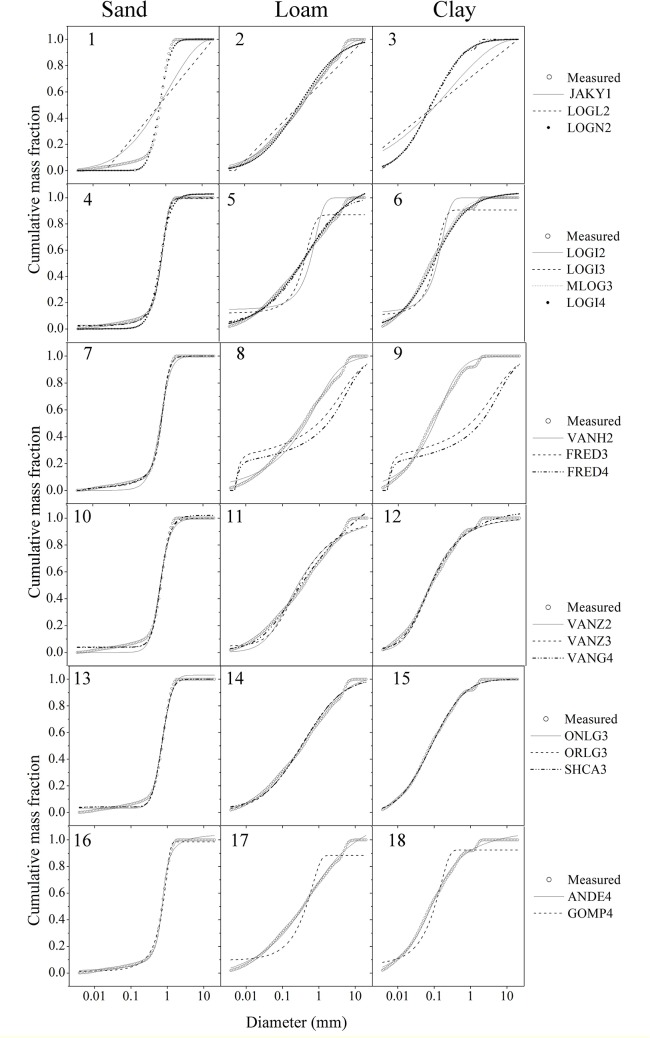
Comparative fit of eighteen mathematical functions to particle size distribution data of different soil samples (n = 3). Sand (Henan soil, 0–20 cm), Loam (Henan soil, 0–20 cm), clay (Hainan soil, 0–20 cm).

An illustration of the comparative fit of the various models to three soil samples of varying textures is shown in Fig 4. It was surprising to find that other than JAKY1 and LOGL2, the models showed no distinct differences in their predicted values at each measurement point for sandy soil (Fig 4.1). This seemed to occur because the PSD has a simpler shape when sand content is high, which can be represented easily by various models. Moreover, this study found that the major difference among the models generally occurred for clay and silt fractions, which could be due to the multivariate shape of the cumulative PSD curve in the size range for such particles. This contrasted with the results of Hwang [9], who found based on SPM PSD data that the major discrepancy in model fitting performance among various PSD models occurred for the silt fraction of soils. The results presented here may be more comprehensive because a larger proportion of the PSD data came from clay fractions.

The LOGN2, MLOG3, LOGI4, VANH2, VANZ2, VANZ3, VANG4, ORLG3, SHCA3, and ANDE4 models consistently provided relatively good fits to all three samples, despite the differences in soil textures (Fig 4). This suggested that these models are suitable for describing detailed soil PSD. The FRED3 and FRED4 (Fig 4.8 and 4.9) models performed poorly for loam and clay soil, which seems to contradict the earlier results that both FRED3 and FRED4 performed better than other models in the *RMSE* and *AIC* analyses. Therefore, it seems that the PSD models that showed good performance for overall soil PSD were not always the most suitable models for individual soil texture classes. The LOGL2 models performed poorly for almost all soils (Fig 4.1–4.3), and therefore these models should be used with caution. Other PSD models had relatively poor performance for at least one soil type, e.g., JAKY1 performed well only for loamy soil, and GOMP4 was suitable only for the sand sample.

Again, as the number of model parameters increased, the extension models LOGI3, MLOG3, and LOGI4 all had better fits than that of the primary two-parameter LOGI2 model. Similarly, the extension models VANZ3 and VANG4 both had smaller *AIC* values than the original VANZ2 model. However with a series of lower *AIC* values, the three-parameter MLOG3 performed better than the four-parameter LOGI4, illustrating that introducing more parameters can appear to improve model fit, even when the model is not necessarily better.

### Effect of soil texture on model performance

**Fig 5:**
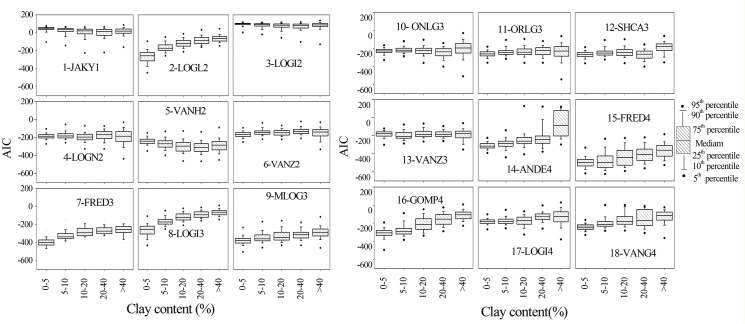
Box plots showing model performance with varying clay content of soils.

**Fig 6:**
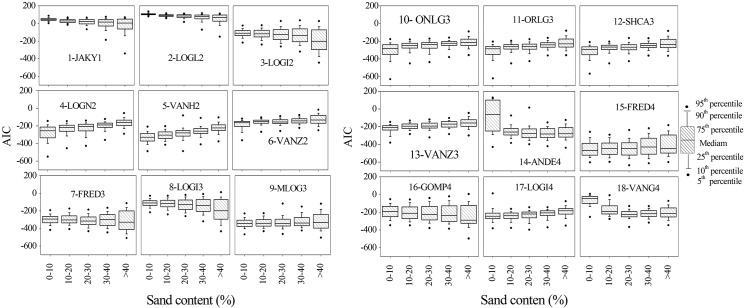
Box plots showing model performance with varying sand content of soils.

**Fig 7:**
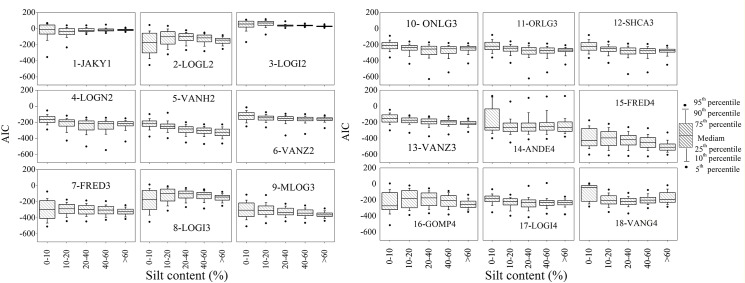
Box plots showing model performance with varying silt content of soils.

**Fig 8:**
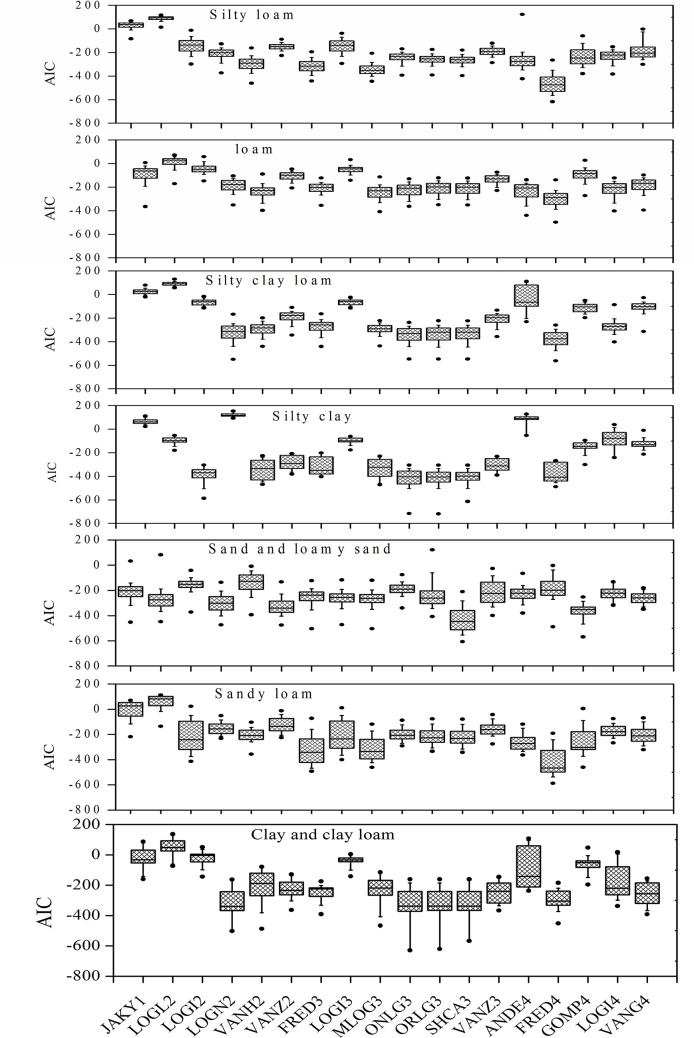
Box plots showing *AIC* for eighteen particle size distribution functions across different soil texture classes.

To examine further the effect of soil texture on model performance, trends in the variation of model *AIC* values were compared. According to the model comparison criteria used here, with increasing in the clay fraction of soils, all models except for VANH2 had poorer fits (Fig 5). Although the rise in *AIC* values was quite limited for some models, i.e., JAKY1, LOGI2, ONLG3, ORLG3, and SHCA3, these trends provided an indication of the relative performance of PSD models with varying clay contents. It was also found that the fits of PSD models (except for LOGI2 and VANG4) declined with increasing sand content. Furthermore, the results presented here indicate that with higher sand content (40%), the *AIC* values for most models had a wider discrete range (Figs 6–8). On this basis, it can be noted that model performance deteriorated as the clay and sand proportions of soils increased, in contrast to previous reports. Hwang found that models performed better as clay content increased [9]. Bah came to similar conclusions after examining seven PSD models with 55 soil samples measured using LDM [26]. The difference in the results presented here may be due to the different data acquisition method and the PSD data density. Usually, with conventional SPM, soil PSD classification data are limited to the resolution of clay size classes, but based on the LDM, it is possible to obtain 19 bins of particle classification data in the clay fraction, which helps the models to depict the soil PSD characteristic distribution curve more accurately. Another contributing factor could be the relatively small and limited sample sizes that Bah used, which covered only two textural classes of clay. The results presented here may be more representative because a larger, more comprehensive soil classification data set was used.

Model performance also varied greatly with silt content (Fig 7). As the silt fraction increased, the models (except for VANG4) had smaller *AIC* values and *AIC* box areas, indicating that the fit of the model improved with increasing silt proportion. Meanwhile, the performance of some models was basically stable, regardless of soil composition, i.e., VANZ2, OLNG3, ORLG3, SHCA3, and VANZ3. These models can therefore be expected to be robust for use under conditions where a large number of samples of different origins, profiles, and landscapes must be analyzed.

*AIC* analysis of models for soils of different clay, silt, and sand contents revealed that the performance of a PSD model can be affected by soil texture. Results indicated that FRED3, MLOG3, and four lognormal models (LOGN2 ONLG3, ORLG3, and SHCA3) performed consistently well for all kinds of soil texture in this study (Fig 8). The FRED4 model had the best fit overall, accounting for most of the smallest *AIC* values for all soils with different textures, except for sand and loamy sand soils. Other studies have previously established that FRED4 is highly suitable for modeling all soil texture classes [26, 8, 9]. The difference in the present results with FRED4, i.e., the finding that it did not perform well for all soil classes, was probably related to the fact that the LDM underestimated the clay fraction. ANDE4 performed well for loam and sandy loam soils. JAKY1, LOGL2, the two logistic models (LOGI2 and LOGI3), and the three van Genuchten models (VANH2, VANZ2, and VANZ3) showed poorer performance for most texture classes. GOMP4 also performed poorly for most soil types; however, for sand and loamy sand, it achieved the second most suitable fit with the PSD data, after SHCA3. LOGI4 seemed to be more appropriate for silty loam, clay, clay loam, loam, and silty clay loam. The performance of VANG4 was only relatively better in silty loam, clay, clay loam, sand, and sandy loam soils.

## Conclusions

The performance of eighteen models in describing the particle size distribution (PSD) of 1013 soil samples has been compared, with the following results. (1) The results of model fitting (*AIC*, adjusted *R* ^*2*^, and *RMSE*) indicated that the FRED4 model provided the best fit for most samples (highest adjusted *R* ^*2*^, lowest *SSE*, and very low *AIC*), (2) The performance of the PSD models was obviously affected by soil texture. Model fit declined as the clay and sand contents of soils increased, except for the VANH2 model. (3) The FRED4 and FRED3 models are preferable for clay, clay loam, loam, loamy sand, sandy clay loam, sandy loam, and silty clay soils. The MLOG3, ONLG3, ORLG3 and SHCA3 models can be used to describe the PSD of a full range of Chinese soils, whereas the remaining models had better performance on one or a few types of soil. (4) This research has referred to previous studies of soil PSD functions and to preliminary studies of several PSD function extension models. Note that these results were based on factors such as small sample size, a wide range of sampling sites, larger differences among soil types, organic matter content, mineral composition, and processes and soil development environment, which led to the relative lowly simulation precision of these PSD models. There is a need to evaluate more alternative models with richer, more comprehensive experimental data, obtained with the LDM from a wider range of soil textures encompassing a greater geographic area, to understand soil particle distributions more completely.

However, the method described here can be expected to be useful under certain conditions. For example, where a larger number of samples of different origins, profiles, and landscapes need to be analyzed, a small number of samples for each soil type can be extracted and analyzed by the SPM and LDM, following which a set of regressive equations can be established to improve the accuracy of LDM PSD results. In this way, error ranges for the LDM can be obtained, and the differences between LDM and SPM can be calibrated. This would provide a basis for choosing a suitable PSD model to estimate soil hydraulic properties.

## Supporting Information

S1 Dataset The RMSE, R2, SSE and AIC for LDM data. (XLS)

S2 Dataset The RMSE, R2, SSE and AIC for PM Data. (XLS)
